# Functional synergy of heteropentameric B subunits underlies virulence in a *Salmonella* A_2_B_5_ toxin

**DOI:** 10.1371/journal.ppat.1013684

**Published:** 2025-11-12

**Authors:** Dongdong Wang, Zhe Chen, Chunyu Xu, Xuyao Jiao, Min Yue, Xiang Gao

**Affiliations:** 1 State Key Laboratory of Microbial Technology, Shandong University, Qingdao, China; 2 Key Laboratory of Systems Health Science of Zhejiang Province, School of Life Science, Hangzhou Institute for Advanced Study, University of Chinese Academy of Sciences, Hangzhou, China.; Indian Institute of Science Education and Research Mohali, INDIA

## Abstract

AB_5_-type toxins are critical virulence factors in bacterial pathogenesis. Despite the identification of various B subunits in AB_5_ toxins across different pathogens, their assembly mechanisms and biological significance remain poorly understood. In this study, we identified and characterized a typhoid toxin-like A_2_B_5_ toxin, designated diarizonae toxin (DiaT), as a key virulence factor in *Salmonella diarizonae*. The DiaT genomic islet encodes two distinct B subunits, PltBd1 and PltBd2, which exhibit unique functional roles. Through genetic and functional analyses, we demonstrate that the heteropentameric assembly of PltBd1 and PltBd2 is essential for cytotoxicity, with our data suggesting PltBd1 facilitates toxin secretion and PltBd2 mediates host cell targeting. Cryo-EM structural analysis of endogenously expressed DiaT reveals a heteropentameric holotoxin with a 3:2 stoichiometry of PltBd1 to PltBd2, potentially stabilized by the PltA subunit. These findings uncover a novel assembly mechanism and synergistic functionality between distinct B subunits, advancing our understanding of the evolutionary diversity and functional complexity of AB_5_ toxins. This work provides new insights into bacterial pathogenesis and highlights potential targets for therapeutic intervention.

## Author summary

AB_5_ toxins are critical virulence factors in bacterial pathogens, but the assembly mechanisms and functional roles of their B subunits remain poorly understood. Our study reveals a novel heteropentameric toxin assembly mechanism in *Salmonella diarizonae*, where two distinct B subunits (PltBd1 and PltBd2) exhibit functional synergy to mediate toxin secretion and host cell targeting. Structural and functional analyses demonstrate that this cooperative interaction enhances cytotoxicity and underscores the evolutionary diversity of AB_5_ toxins. Our findings provide new insights into bacterial pathogenesis and highlight potential therapeutic targets for combating infections caused by AB_5_ toxin-producing pathogens.

## Introduction

AB_5_-type toxins, a class of secreted protein toxins, are key virulence factors in bacterial pathogens [1–11]. In *Salmonella enterica* serovar Typhi, typhoid toxin (TT)—a unique A_2_B_5_ toxin—plays a central role in the pathogenesis of typhoid fever [12–14]. TT consists of two active A subunits, CdtB and PltA, and a pentameric B subunit, PltB [15–17]. CdtB is a deoxyribonuclease that induces DNA damage in host cells [10,18–21], while PltA functions as an ADP-ribosyltransferase with unknown targets [20]. PltB specifically binds to glycans, facilitating the transport of CdtB and PltA [15,21–27]. During infection, *S.* Typhi resides within a *Salmonella*-containing vacuole (SCV), where it synthesizes and assembles TT [17,20,28]. PltB binds to glycan receptors on the SCV membrane [17,23] and packages TT into vesicles for extracellular release [24]. Released toxins then invade target cells by binding to surface glycan receptors through PltB [12,17,23,26]. Once inside host cells, CdtB induces cell cycle arrest and apoptosis [12,17,23].

Recently, A_2_B_5_-like typhoid toxins have been identified in typhoidal and non-typhoidal *Salmonella* strains [2,29–34]. Multiple B subunits have been described in *S.* Typhi [2,30] and other non-typhoidal *Salmonella* strains [35]. In *S.* Typhi, the *pltB* and *pltC* genes, encoding homologous B subunits, are located in separate genomic loci [30]. Both PltB and PltC can form homopentamers [30], but heteropentamer formation between PltB and PltC is not possible due to electrostatic repulsion at their interface [31]. This results in two distinct toxin variants, PltB-TT and PltC-TT, in *S.* Typhi. *In vitro* copurification of PltB and ArtB, a second B subunit in *Salmonella* Javiana, suggests that heteropentameric binding subunits may occur [35], thus enhancing the diversity of A_2_B_5_ toxins. However, the mechanisms and biological significance of these multiple B subunit assemblies remain unclear.

*Salmonella diarizonae*, a subspecies of *S. enterica*, primarily infects reptiles, especially snakes. Human infections often occur through direct contact with reptiles, their meat, or water contaminated by the pathogen [36,37]. In the United States, approximately 74,000 annual cases of *Salmonella* infection are linked to reptile exposure [38,39]. *S. diarizonae* was also detected in sheep and migratory birds, posing risks via contaminated meat [40–43]. While *S. diarizonae* typically causes self-limiting gastroenteritis [44], it can also lead to more severe conditions, such as maxillary sinusitis [36], bacteremia [45], and cervical lymphadenitis [44]. Children under 10 and immunocompromised individuals are particularly vulnerable to severe outcomes like sepsis and meningitis [46,47]. However, the pathogenic mechanisms of *S. diarizonae* remain poorly understood.

Here, we identify a genetic locus encoding a typhoid-like toxin, diarizonae toxin (DiaT), in *S. diarizonae*. Unlike *S.* Typhi and *S.* Javiana, where the genes encoding two B subunits are located in separate loci, *S. diarizonae* houses both B subunit genes (*pltBd1* and *pltBd2*) within the same genomic island [27,30], indicating a novel gene arrangement for typhoid-like toxins. We show that the cytotoxicity of *S. diarizonae* to host cells depends on the heteropentameric assembly of PltBd1 and PltBd2. PltBd1 contributes to toxin secretion from the SCV, while PltBd2 is involved in toxin endocytosis into host cells. Structural analysis reveals that the B subunits stabilize each other through polar interactions, with a 3:2 ratio of PltBd1 to PltBd2 in the heteropentameric holotoxin, potentially governed by PltA. The discovery of DiaT, which provides the first structural elucidation of a heteropentameric A_2_B_5_ toxin, redefines the diversity of AB_5_ toxins and reveals a novel synergistic mechanism between distinct B subunits. These insights could pave the way for targeted therapies against DiaT-related diseases.

## Results

### Identification of a virulence-associated typhoid toxin-like A_2_B_5_ toxin in *S. diarizonae*

**Fig 1:**
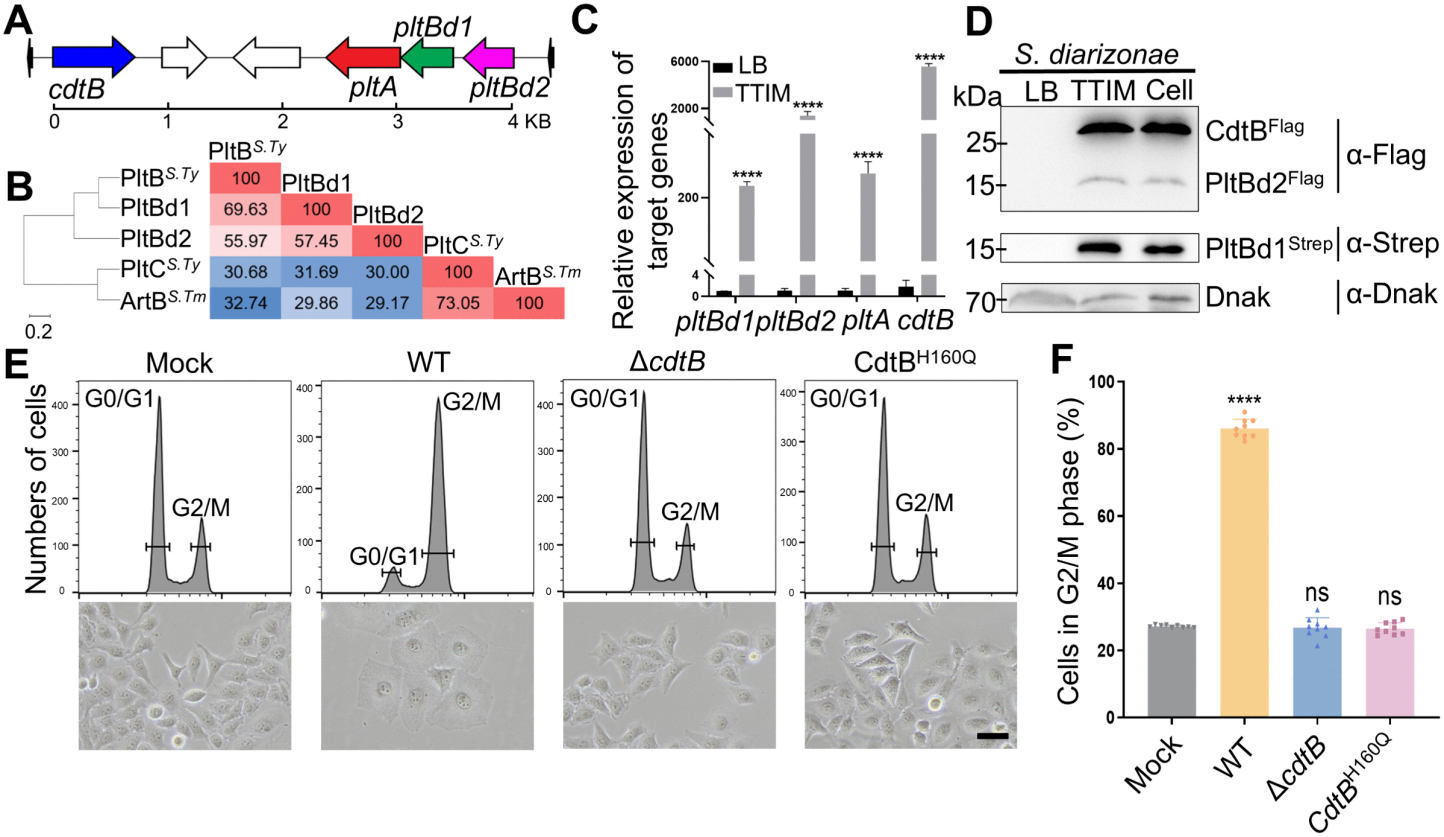
Diarizonae toxin (DiaT) is a typhoid toxin-like virulence factor in *S. diarizonae.* **(A)** Schematic of the DiaT genomic island, encoding CdtB, PltA, PltBd1 and PltBd2. Colored arrows represent genes *cdtB* in bright blue encoding CdtB, a cytolethal distending toxin-like component, *pltA* in red encoding PltA, an ADP-ribosyltransferase, *pltBd1* in green and *pltBd2* in bright violet encoding two PltB, the receptor-binding proteins. **(B)** Phylogenetic tree and homology comparison of PltBd1 and PltBd2 with known B subunits of other AB_5_ toxins, demonstrating evolutionary proximity to PltB and divergence from ArtB. **(C)** RT-qPCR analysis of transcript levels of diarizonae toxin genes in LB (black bar) and typhoid toxin-inducing medium (TTIM) (gray bar). Data in experiment C represents the mean ± standard deviation (s.d.) from three biological replicates (n = 3) with triplicate measurements per replicate. A paired two-tailed *t*-test was used to analyze the statistical significance; ****p < 0.0001. **(D)** Western blot analysis confirming DiaT expression in TTIM and in infected human cells, but not in LB medium. DnaK expression was used as a control, and kDa indicates the protein size. Experiment D was conducted at least three times with consistent results. **(E-F)** Cytotoxicity assays showing the loss of cytotoxicity in Δ*cdtB* and CdtB^H160Q^ strains towards NCM460 cells (MOI = 10). The proportion of cells in G2/M, reflecting the toxic activity of CdtB, following infection with wild-type *S. diarizonae* and its mutant strains, was determined by flow cytometry. Data in experiment F represents the mean ± s.d. from three biological replicates (n = 3) with triplicate measurements per replicate. An unpaired two-tailed *t-*test was used to analyze the statistical significance; ns (not significant), ****p < 0.0001. Scale bar: 50 µm.

Genome sequencing and assembly of a clinical *S. diarizonae* isolate revealed a genomic island encoding homologs of typhoid toxin (TT) genes, including *cdtB*, *pltA*, and two *pltB* variants (*pltBd1* and *pltBd2*) (Fig 1A). The arrangement of *cdtB* and *pltA* is conserved relative to the TT islet, and their respective protein products, CdtB and PltA, exhibit high sequence identity with TT components: 93% for CdtB and 78% for PltA. Notably, the active catalytic site of CdtB and the cysteine residues responsible for forming the disulfide bond between CdtB and PltA are conserved in the putative *S. diarizonae* toxin, underscoring its structural and functional similarity to TT (S1A Fig).

Unlike the TT genomic organization, the *S. diarizonae* toxin island features two distinct *pltB* homologues (*pltBd1* and *pltBd2*) located within the same genomic cluster but separated by a 117-base pair interval (Fig 1A). Sequence alignment showed that PltBd1 shares 69%, 32%, and 30% identity with PltB^*S.Ty*^, PltC^*S.Ty*^, and ArtB^*S.Tm*^, respectively, while PltBd2 shares 56%, 30%, and 29% identity with these proteins (Fig 1B). Phylogenetic analysis positioned PltBd1 and PltBd2 closer to PltB^*S.Ty*^, highlighting their evolutionary relationship (Fig 1B).

Expression analysis revealed that the transcription and expression of the putative toxin genes occur exclusively in typhoid toxin-inducing medium (TTIM), but not in LB medium (Figs 1C, 1D, and S1B). TTIM, a previously described chemically defined growth medium with low Mg^2+^ to mimic the SCV environment and activate Mg^2+^-responsive promoters, was employed to promote toxin secretion [48]. Moreover, toxin expression can be detected intracellularly in normal human colon mucosal epithelial cells (NCM460) following bacterial infection. This regulatory pattern is consistent with the intracellular expression of TT.

Functional assays further confirmed that the cytotoxic activity of *S. diarizonae* relies on CdtB. Deletion of *cdtB* (Δ*cdtB*) or its catalytic mutation (CdtB^H160Q^) abolished cytotoxicity in infected cells (Fig 1E, 1F). Collectively, these findings establish that the identified genomic island encodes a functional A_2_B_5_-like toxin critical for *S. diarizonae* virulence, herein designated as diarizonae toxin (DiaT).

### Heteropentameric B subunits of DiaT are indispensable for the cytotoxicity of *S. diarizonae*

In AB_5_-type toxins, B subunits assemble into pentamers to facilitate toxin delivery and cytotoxicity. Uniquely, *S. diarizonae* encodes two distinct B subunits (PltBd1 and PltBd2) within the same toxin locus. To determine their roles, we evaluated the cytotoxicity of *S. diarizonae* strains lacking either *pltBd1*, *pltBd2*, or both. Surprisingly, none of these mutants exhibited cytotoxic effects in infected NCM460 cells (S1D Fig). Further analysis revealed that *pltBd1* deletion impaired *pltBd2* transcription and expression (S1E, S1F Fig).

**Fig 2:**
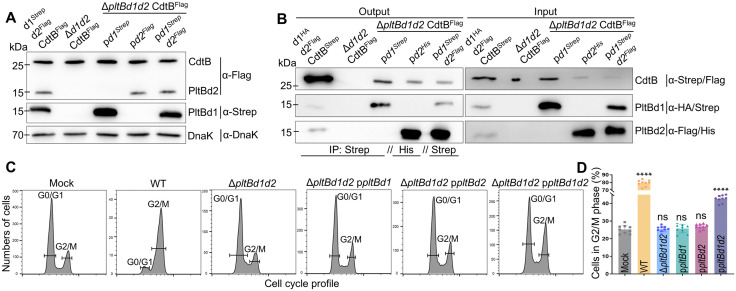
PltBd1/PltBd2 heteropentameric holotoxin is required for *S. diarizonae* cytotoxicity. **(A)** Expression levels of PltBd1 and PltBd2 in the wild-type and complemented *S. diarizonae* strains were assessed via Western blot after growth in TTIM medium. **(B)** Co-immunoprecipitation (Co-IP) analysis of holotoxins purified from wild-type and complemented strains. Complementation with either *pltBd1* or *pltBd2* alone resulted in homopentameric B subunits, while co-complementation enabled the assembly of a heteropentameric holotoxin. Experiments A-B were conducted at least three times with consistent results. **(C-D)** Cytotoxicity assays on NCM460 cells (MOI = 10) revealed that homopentameric holotoxins failed to induce cytotoxicity, while heteropentameric holotoxins partially restored cytotoxicity. The proportion of cells in G2/M, reflecting the toxic activity of CdtB, following infection with wild-type *S. diarizonae* and its mutant strains, was determined by flow cytometry. Data in histogram represents the mean ± s.d. of three biological replicates (n = 3), each performed in triplicate. Statistical analysis was performed using unpaired two-tailed *t*-tests; ns, not significant, ****p < 0.0001.

To resolve the functional significance of the PltBd1 and PltBd2 subunits in *S. diarizonae*, we deleted both genes and subsequently complemented the double-deletion strain with either *pltBd1* or *pltBd2* individually. Western blot analysis confirmed that the expression levels of PltBd1 and PltBd2 in the complemented strains were comparable to those in the wild-type strain grown in TTIM medium (Figs 2A and S1C). Individual complementation with either *pltBd1* or *pltBd2* led to the assembly of holotoxins with homopentameric B subunits (Fig 2B), yet neither restored the cytotoxicity of *S. diarizonae* (Fig 2C, 2D). However, co-complementation with both *pltBd1* and *pltBd2* facilitated the formation of a PltBd1/PltBd2 heteropentameric holotoxin (Fig 2B), partially restoring cytotoxicity (Fig 2C, 2D). The attenuated cytotoxicity likely results from PltBd1 overexpression in the complemented strain, which may increase formation of PltBd1 homopentamers and impair the assembly of functional heteropentameric holotoxins. These results demonstrate that both PltB subunits are essential for forming a functional heteropentameric holotoxin, underscoring their critical role in the pathogenicity of *S. diarizonae* and the unique assembly mechanism of DiaT.

### Endogenously expressed PltBd1 and PltBd2 form a heteropentameric holotoxin during infection

**Fig 3:**
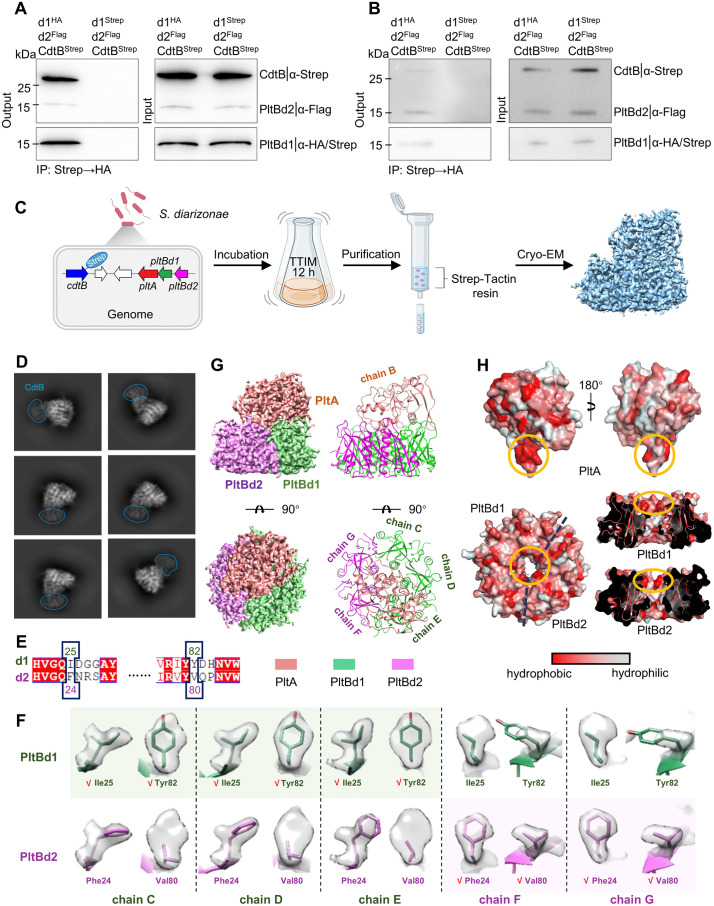
The architecture of DiaT holotoxin. **(A-B)** Co-IP analysis demonstrates the assembly of the PltBd1/PltBd2 heteropentameric holotoxin under TTIM culture conditions (A) and in infected host cells **(B)**. Experiments were performed at least three times with consistent results. **(C)** Workflow for *in situ* purification and structural determination of the heteropentameric holotoxin, employing Strep-Tactin affinity chromatography and gel filtration followed by cryo-EM data collection. Created with Biorender.com. https://BioRender.com/o05m2ra **(D)** Representative 2D class averages from cryo-EM analysis, illustrating visible CdtB density and its connection to PltA. **(E)** Key amino acid differences in cryo-EM maps used to differentiate PltBd1 and PltBd2. **(F)** Close-up views of cryo-EM electron density maps highlighting side-chain differences: Ile25 and Tyr82 (PltBd1) versus Phe24 and Val80 (PltBd2). **(G)** Two cryo-EM views of the heteropentameric holotoxin showing the pseudo-symmetric 3:2 arrangement of PltBd1 and PltBd2 subunits. **(H)** Two perspectives of PltA’s amphipathic C-terminal tail, including a 180° rotation (top) and the hydrophobic inner channels formed by the heteropentameric B subunits (bottom) with the close-up views of the channel.

To confirm whether *S. diarizonae* assembles a PltBd1/PltBd2 heteropentameric holotoxin during infection, we generated a strain with *in situ* tagged DiaT components. Co-immunoprecipitation (Co-IP) and Western blot analysis revealed that *S. diarizonae* forms the heteropentameric holotoxin under TTIM conditions and within infected host cells (Fig 3A, 3B). Additionally, recombinant co-expression of PltBd1, PltBd2, PltA, and CdtB in *Escherichia coli* similarly demonstrated the presence of the heteropentameric holotoxin (S1G, S1H Fig).

### Pseudo-symmetric 3:2 assembly of PltBd1 and PltBd2 in the DiaT holotoxin

To elucidate the structural assembly of PltBd1 and PltBd2 within the DiaT holotoxin, we introduced a C-terminal Strep-tag on CdtB in the *S. diarizonae* genome. DiaT was purified from cultures grown in TTIM medium using Strep-Tactin affinity purification and gel filtration, followed by cryo-EM analysis (Fig 3C). To better distinguish two B subunits sharing 57.45% sequence identity, we collected over 5,376 cryo-EM images and analyzed over one million protein particles. Ultimately, we obtained only a single class of high-resolution cryo-EM map for the holotoxin, at a resolution of 2.32 Å (S2 Fig). Despite clear visualization of CdtB in 2D classifications (Fig 3D), its flexible linkage to PltA, mainly via a single disulfide bond, caused a weakened signal, leading to its absence in the final 3D reconstruction.

To distinguish PltBd1 from PltBd2 in the cryo-EM map, we identified residues with distinct structural features between PltBd1 and PltBd2 (Fig 3E). Specifically, Ile25 and Tyr82 in PltBd1 corresponded to Phe24 and Val80 in PltBd2, respectively, providing clear markers for differentiation (Fig 3F). Detailed analysis of electron density maps revealed a pseudo-symmetric arrangement of the pentameric B subunits, comprising a 3:2 ratio of PltBd1 to PltBd2. Three tightly packed PltBd1 subunits were connected with dimeric PltBd2 subunits to form the heteropentamer in the holotoxin structure (Fig 3G).

### The overall structure of DiaT holotoxin

Building on the characterization of the unique 3:2 assembly of pentameric B subunits, we resolved the cryo-EM structure of the endogenously expressed DiaT holotoxin from *S. diarizonae*. The holotoxin consists of a pentameric B subunit arrangement supporting a single PltA subunit, with the amphipathic C-terminal tail of PltA extending into the central pore formed by the B subunits (Fig 3G). The hydrophobic face of the C-terminal tail stabilizes its integration into the pore, while the amphipathic nature ensures compatibility with surrounding molecules (Fig 3H). This structural configuration mirrors that of the PltA subunit in typhoid toxin, underscoring the essential role of the C-terminal tail in mediating interactions with the B subunit pentamer and maintaining the holotoxin’s overall stability.

To validate the structural insights from the cryo-EM map of the endogenously expressed DiaT holotoxin, we recombinantly expressed PltBd1 homopentameric holotoxin, PltBd2 homopentameric holotoxin, and PltBd1/PltBd2 heteropentameric holotoxin in *E. coli*. Cryo-EM analysis resolved these structures at resolutions of 3.05 Å, 2.61 Å, and 2.94 Å, respectively (S3–S5 Figs). Structural analysis confirmed consistent and distinct amino acid differences that enable the differentiation of PltBd1 and PltBd2 in the endogenous DiaT holotoxin (S6A Fig), further reinforcing their reliability as markers for B subunit identification. Notably, the heteropentameric holotoxin purified from *E. coli* exhibited an identical composition and assembly to the endogenous DiaT holotoxin purified from *S. diarizonae* (S6B Fig). Moreover, despite successful recombinant expression of homopentamers, *S. diarizonae* predominantly forms holotoxins with heteropentameric B subunits, reflecting its natural assembly mechanism when both PltBd1 and PltBd2 are co-expressed. Comparative structural analysis revealed that PltB^*S.Ty*^, PltBd1, and PltBd2 share a highly conserved structural framework with key features, including two disulfide bond pairs and a critical serine residue, maintaining identical spatial positions (S7 Fig).

### Structural basis of heteropentameric B subunit assembly in DiaT

**Fig 4:**
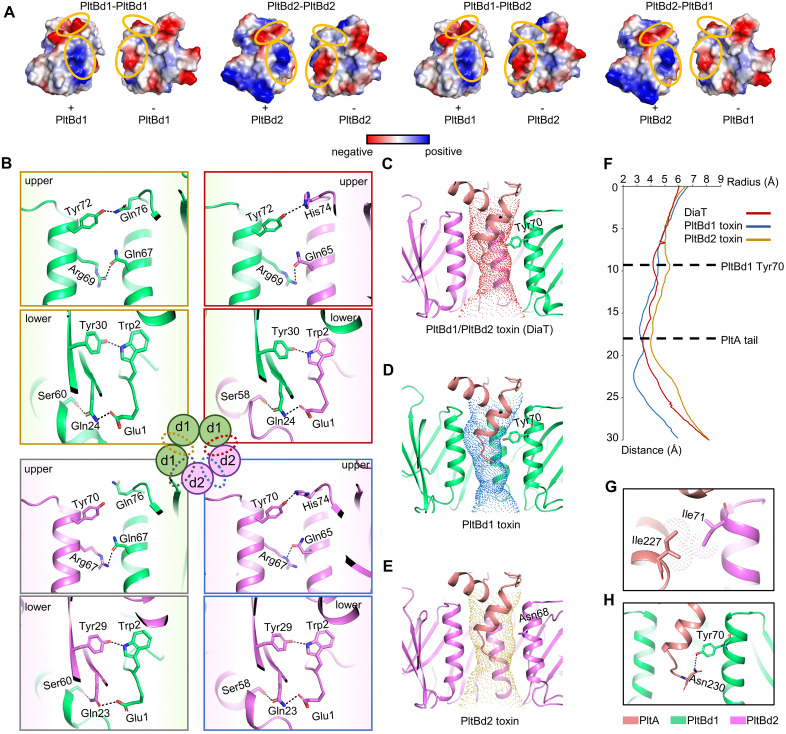
Molecular mechanisms of DiaT holotoxin subunit interactions. **(A)** Surface charge distribution of interaction surfaces for PltBd1-PltBd1, PltBd2-PltBd2, PltBd1-PltBd2, and PltBd2-PltBd1. Blue represents positive charge, and red represents negative charge. **(B)** Key molecular interactions at the B subunit interfaces, including Arg69/Tyr72 in PltBd1 and Arg67/Tyr70 in PltBd2, alongside conserved lower-interface residues Gln24/23, Glu1, Ser60/58, Tyr30/29, and Trp2. **(C-E)** Comparative analysis of the central channel radius in the PltBd1/PltBd2 heteropentamer **(C)**, PltBd1 homopentamer **(D)**, and PltBd2 homopentamer **(E)**, revealing contraction near Tyr70 in PltBd1. **(F)** Pore radius measurements for PltBd1/PltBd2, PltBd1, and PltBd2 pentamers, with key residues marked. The black dotted lines show the positions of Tyr70 in PltBd1 and the end of the PltA C-terminus in the channels, respectively. **(G)** Stable hydrophobic interaction between Ile227 of PltA and Ile71 of PltBd2. **(H)** Key polar interaction between Tyr70 in PltBd1 and Asn230 in PltA, contributing to the contracted pore and holotoxin stability.

To elucidate the assembly mechanism of heteropentameric B subunits in DiaT, we analyzed the contact surfaces between subunits. Both PltBd1 and PltBd2 exhibit similar surface electrostatic potentials, particularly at their interaction sites (Fig 4A). Surface charge mapping revealed a consistent distribution of significant polar charges across the subunits, especially in the upper and central regions where inter-subunit interactions occur (Fig 4A). This concentrated distribution of polar interactions facilitates the stable assembly of the heteropentamer.

Detailed inspection of the interaction interfaces revealed distinct features. On the upper interface, PltBd1 harbors Arg69 and Tyr72, while PltBd2 features Arg67 and Tyr70 (Fig 4B). Despite minor variations at the Tyr interaction residues (Gln76 in PltBd1 and His74 in PltBd2), both pairs form stable polar interactions (Fig 4B). The lower interface interactions are nearly identical and primarily involve conserved residues such as Gln24/23, Glu1, Ser60/58, Tyr30/29, and Trp2, ensuring robust connections across diverse subunit interfaces (Fig 4B). Importantly, site-directed mutagenesis of these interfacial residues (PltBd1: E1, W2, Q24, Y30, S60, N67, R69, Y72, N76; PltBd2: E1, W2, Q23, Y29, S58, N65, R67, Y70, H74) demonstrated their essential role in heteropentamer assembly while maintaining normal expression (S8A Fig). Interestingly, PltB pentamerization occurred independently of PltA/CdtB (S8B Fig), demonstrating an intrinsic self-assembly property. This autonomy is driven by complementary surface electrostatics and specific molecular interactions.

### Complementary roles of PltBd1 and PltBd2 in stabilizing PltA in DiaT

Given the pseudo-symmetric arrangement of the PltBd1/PltBd2 heteropentamer, we investigated how interactions between PltA and the B pentamer influence the assembly and stability of the holotoxin. Our analysis revealed that PltA’s C-terminal tail inserts into the central pore formed by the pentameric B subunits, anchoring the interaction. While PltBd1 and PltBd2 share similar structural scaffolds for binding PltA, their specific interactions with PltA differ. A localized distortion in the central pore was observed near Tyr70 in PltBd1, where the bulky phenolic side chain creates steric hindrance, causing contraction at this site (Fig 4C).

Pore radius measurements confirmed that the PltBd1 homopentamer exhibits the smallest pore radius with significant distortion, while the PltBd2 homopentamer displays a larger and straighter pore (Fig 4D, 4E). These findings suggest that the specific positioning of Tyr70 in PltBd1 is responsible for the pore contraction in the heteropentamer, which enhances compatibility with the C-terminal tail of PltA (Fig 4F).

Further analysis of PltA’s interactions with distinct B subunits identified a critical hydrophobic interaction between Ile227 of PltA and Ile71 of PltBd2 (Fig 4G). This interaction is weaker in the PltBd1 homopentamer due to the shorter side chain of Val73 in PltBd1 (S9 Fig). Additionally, Tyr70 in PltBd1 forms stable polar interactions with Asn230 in PltA, contributing to the narrow, contracted pore structure (Fig 4H), a feature absents in the PltBd2 homopentamer (Fig 4G).

These structural insights highlight the complementary roles of PltBd1 and PltBd2 in stabilizing the heteropentameric holotoxin. The stronger hydrophobic interactions between PltBd2 and PltA, along with additional polar interactions involving PltBd1 and PltA, establish a finely balanced assembly that facilitates the stable accommodation of PltA’s C-terminal tail by the heteropentameric B subunits (S9 Fig).

### The role of PltBd2 in mediating toxin endocytosis to drive cytotoxicity

During *S.* Typhi infection, secretion and endocytosis of typhoid toxin rely on the PltB subunit, where Ser35 plays a key role in glycan receptor binding on the SCV inner surface and target cell membranes. These interactions are crucial for toxin release and subsequent target cell entry. Infection assays and cryo-EM analyses have revealed that DiaT adopts a heteropentameric structure essential for the cytotoxicity of *S. diarizonae*. Therefore, we aim to elucidate the functional significance underlying the necessity of this heteropentameric conformation of B subunits for DiaT cytotoxicity.

**Fig 5:**
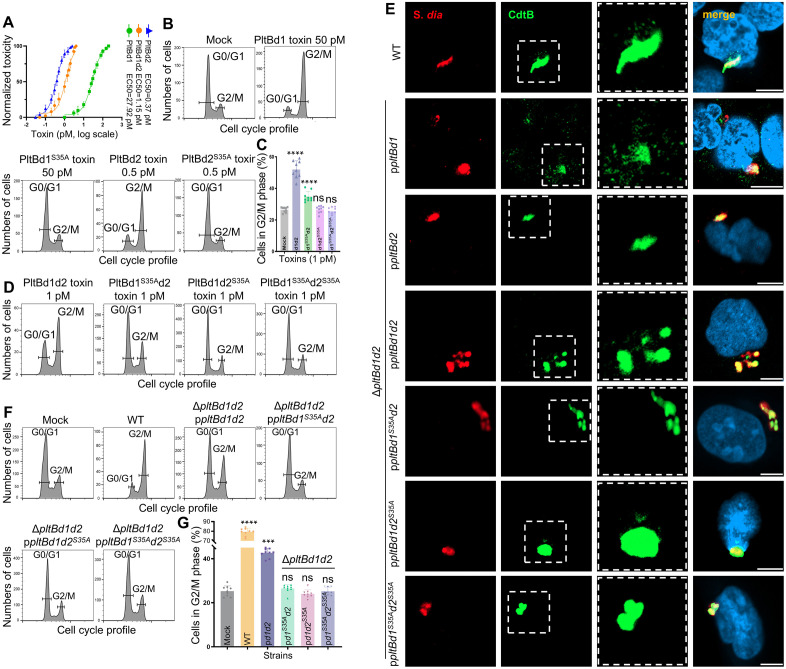
Distinct and synergistic functions of PltBd1 and PltBd2 in toxin secretion and toxin endocytosis. **(A)** EC50 values for the cytotoxicity of purified holotoxins composed of PltBd1 homopentamers (green line), PltBd2 homopentamers (blue line), or PltBd1/PltBd2 heteropentamers (orange line) were determined using cultured NCM460 cells. **(B)** Cytotoxicity of wild-type and S35A-mutated PltBd1/PltBd2 homopentameric holotoxins was measured in NCM460 cells. **(C-D)** Cytotoxicity of wild-type and S35A-mutated PltBd1/PltBd2 heteropentameric holotoxins was measured in NCM460 cells. Data in histogram represents the mean ± s.d. of three biological replicates (n = 3), each performed in triplicate. **(E)** Immunofluorescence imaging of toxin transport carriers in NCM460 cells infected with various *S. diarizonae* strains expressing FLAG-tagged CdtB, which was conducted with three biological replicates. Discrete CdtB puncta, representing toxin release from SCVs, were detected using a mouse anti-FLAG monoclonal antibody (green). *Salmonella* and NCM460 were visualized using a rabbit anti-*Salmonella* antibody (red), and DAPI nuclear staining (blue), respectively. Confocal laser scanning microscopy revealed toxin localization within the host cells. Scale bar: 10 µm. **(F-G)** Cytotoxicity assays performed on NCM460 cells infected with *S. diarizonae* strains complemented with heteropentameric holotoxins (MOI = 10). Data in histogram represents the mean ± s.d. of three biological replicates (n = 3), each performed in triplicate. Statistical significance was analyzed using unpaired two-tailed *t*-tests; ns, not significant, ***p < 0.001, ****p < 0.0001.

To delineate the roles of PltBd1 and PltBd2 in toxin secretion and endocytosis, we initially performed intoxication assays using purified holotoxins composed of either homopentameric or heteropentameric assemblies on cultured NCM460 cells. All holotoxins demonstrated cytotoxic activity, but the PltBd2 homopentamer exhibited the highest potency, approximately threefold greater than the PltBd1/PltBd2 heteropentameric toxin (DiaT) and nearly 75-fold greater than the PltBd1 homopentamer (Fig 5A). Further assays using S35A mutants of PltBd1 and PltBd2 homopentamers confirmed that the S35A mutation completely abolished toxin cytotoxicity, underscoring the essential role of Ser35 in glycan receptor binding and subsequent activity (Fig 5B).

Interestingly, while the heteropentameric holotoxin PltBd1^S35A^d2 retained limited residual activity, the PltBd1d2^S35A^ mutant was entirely inactive. These findings imply a potential dominant role of PltBd2 in facilitating toxin endocytosis into host cells and DiaT cytotoxicity while highlighting the strict dependence on Ser35 for glycan receptor engagement (Fig 5C, 5D).

### The role of PltBd1 in toxin secretion from SCV of infected cells

Despite its potent intoxication potential (Fig 5A), the PltBd2 homopentameric holotoxin failed to restore cytotoxicity in the *pltBd1* and *pltBd2* double deletion strain complemented with *pltBd2* alone (Fig 2B, 2C). This suggests that PltBd2 homopentamers are likely unable to mediate extracellular secretion. To explore this, we examined toxin carrier formation in cells infected with various *S. diarizonae* strains expressing FLAG-tagged CdtB. Immunofluorescence microscopy revealed discrete CdtB puncta, released from the SCV, in cells infected with the wild-type strain and complemented strains Δ*pltBd1d2*::p*pltBd1* and Δ*pltBd1d2*::p*pltBd1d2* (Figs 5E and S10A). In contrast, puncta were absent in cells infected with the Δ*pltBd1d2*::p*pltBd2* strain (Fig 5E), despite comparable CdtB expression in the wild-type (Fig 2A). These findings indicate that PltBd1 contributes to package DiaT into vesicle carrier intermediates and enabling extracellular secretion.

To further dissect the distinct contributions of PltBd1 and PltBd2 to the PltBd1/PltBd2 heteropentameric holotoxin, we infected cultured NCM460 cells with *S. diarizonae* Δ*pltBd1d2* mutants complemented with constructs encoding Δ*pltBd1d2*::p*pltBd1*^*S35A*^*d2*, Δ*pltBd1d2*::p*pltBd1d2*^*S35A*^, or Δ*pltBd1d2*::p*pltBd1*^*S35A*^*d2*^*S35A*^. Co-immunoprecipitation confirmed the formation of heteropentameric holotoxins in all complemented strains (S10B Fig). The strains complemented with *pltBd1*^*S35A*^*d2* and *pltBd1*^*S35A*^*d2*^*S35A*^, which formed the PltBd1/PltBd2 heteropentamer, failed to secrete the toxin (Fig 5E) and exhibited no cytotoxicity (Fig 5F, 5G). This indicates that the S35A mutation in PltBd1 disrupts glycan binding, impairing toxin secretion. In contrast, the strain complemented with *pltBd1d2*^*S35A*^ successfully formed the heteropentameric holotoxin (S10B Fig) and secreted it (Fig 5E), but it was non-toxic (Fig 5F, 5G), highlighting the essential role of PltBd2 in toxin endocytosis into host cells.

To address potential concerns regarding cell type specificity, we extended our assays to human colon adenocarcinoma cell line HT-29 (S11 Fig). Compared to NCM460 cells, HT-29 models demonstrated greater toxin tolerance but a consistent pattern of cytotoxicity, with PltBd2 displaying maximal cytotoxicity (S11A Fig). Functional PltBd2 is required for cytotoxicity, as its activity alone retained partial toxicity in intoxication assay (S11B, S11C Fig). In contrast, while PltBd1 enables toxin secretion (S11D Fig), the S35A mutation in PltBd2 abolished cytotoxicity in Δ*pltBd1d2* complemented strains (S11E, S11F Fig). Consistent observations in both cell lines suggest that PltBd1 facilitates toxin secretion from the SCV of infected cells, while PltBd2 contributed to target cell entry. The S35A mutation in either subunit disrupts glycan receptor binding, impairing these distinct functions.

### Carbohydrate-binding specificity of PltBd1 and PltBd2

The carbohydrate-binding specificity of B subunits in AB_5_ toxins is critical for their function. In typhoid toxin, the PltB subunit specifically binds carbohydrates terminating in N-acetylneuraminic acid (Neu5Ac) via its lateral carbohydrate-binding site at Ser35 [15]. By contrast, PltC and ArtB recognize both Neu5Ac- and N-glycolylneuraminic acid (Neu5Gc)-terminated carbohydrates, but an additional tyrosine residue within their lateral binding pockets confers selective specificity for Neu5Gc [27,31].

**Fig 6:**
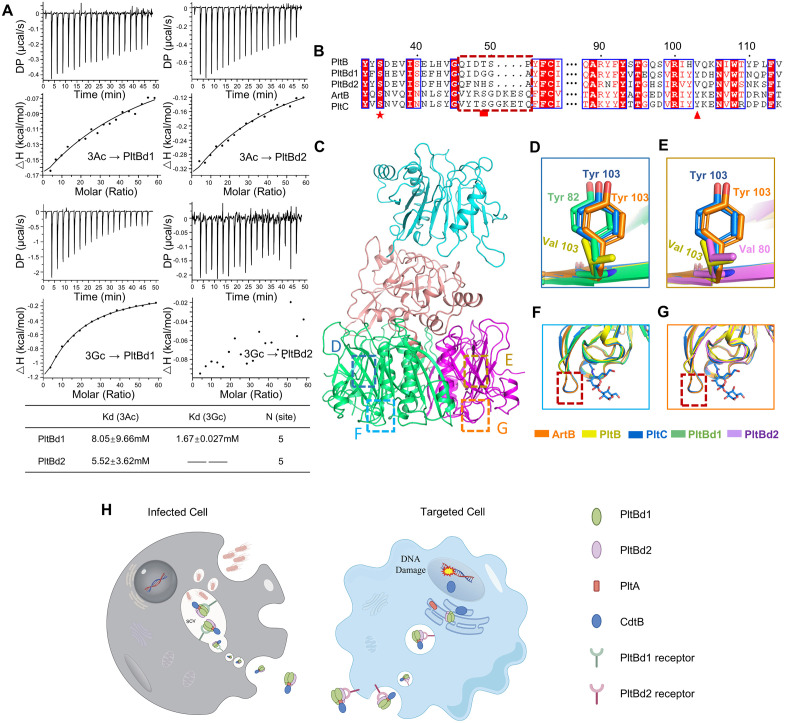
Structural and functional basis for distinct carbohydrate-binding specificities of PltBd1 and PltBd2. **(A)** Isothermal titration calorimetry (ITC) assays comparing the binding affinities of PltBd1 and PltBd2 pentamers for Neu5Ac-terminated glycans (3Ac) and Neu5Gc-terminated glycans (3Gc). Experiments were repeated at least three times with consistent results. **(B)** Amino acid sequence alignment of PltBd1, PltBd2, and their homologous B subunits. Conserved residues are highlighted in red. The pentacle marks the conserved lateral carbohydrate-binding site (Ser35). The triangle indicates the Tyr103 residue in PltBd1, PltC, and ArtB. The dashed frame highlights the spoon-shaped basal glycan-binding site present in PltC and ArtB but absent in PltBd1, PltBd2, and PltB. **(C)** Cryo-EM structure of DiaT holotoxin. Dashed rectangle boxes locate PltBd1 and PltBd2 subunits analyzed in D-G. **(D-G)** Comparison of structural features in PltBd1, PltBd2, and homologous B subunits. **(D-E)** Close-up structural views of the lateral carbohydrate-binding sites, generated by structural alignment to ArtB (PDB: 5WHU), PltB-Neu5Ac complex (PDB: 5WHT), and PltC-Neu5Ac complex (PDB: 7EE4). PltBd1 features a Tyr103 residue **(D)**, while PltBd2 has a valine residue at the same position **(E)**. **(F-G)** Structural comparison of the PltC spoon-shaped basal carbohydrate-binding site (highlighted by a rectangle) with the same position in ArtB (PDB: 5WHU), PltB-Neu5Ac complex (PDB: 5WHT), PltC-Neu5Ac complex (PDB: 7EE4). **(H)** Schematic diagram depicting the indispensable role of PltBd1 and PltBd2 in the cytotoxicity of *S. diarizonae*. The diarizonae toxin comprises a trimeric PltBd1 and dimeric PltBd2, forming a heteropentameric B subunit assembly. Our data suggest that PltBd1 may promote toxin secretion from infected cells, while PltBd2 might mediate host cell targeting. The illustration rendering portion of this work was supported by Figdraw (https://www.figdraw.com/).

To delineate the carbohydrate-binding specificities of PltBd1 and PltBd2, we conducted isothermal titration calorimetry (ITC) assays to measure their affinities for Neu5Ac- and Neu5Gc-terminated versions of a representative glycan (3Ac and 3Gc; Neu5Ac*α*2-3G*a*l*β*1-4Glc and Neu5Gc*α*2-3Gal*β*1-4Glc, respectively). The synthetic glycans were selected based on critical binding features identified by typhoid toxin-specific glycan microarray screening: preferential recognition of α2-3-linked sialic acid and Gal*β*1-4Glc [15]. Both PltBd1 and PltBd2 pentamers exhibited binding to Neu5Ac-terminated glycans. The lower average dissociation constant exhibited by PltBd2 suggests a higher affinity for these glycans than PltBd1. However, only PltBd1 displayed substantial binding to Neu5Gc-terminated glycans (Fig 6A).

### Structural basis for substrate recognition by PltBd1 and PltBd2

Consistent with the ITC findings, sequence analyses identified structural differences underlying the distinct glycan-binding specificities of PltBd1 and PltBd2. Both subunits share a conserved lateral carbohydrate-binding site at Ser35, essential for glycan interaction (Fig 6B, pentacle). However, PltBd1, similar to PltC and ArtB, contains a Tyr103 residue at this site (Fig 6B, triangle and 6D). Structural modeling based on the PltC-Neu5Gc complex (PDB:7EE5) revealed that the phenolic hydroxyl group of Tyr103 forms a 2.9 Å hydrogen bond with the terminal hydroxyl of Neu5Gc (S12 Fig), providing a molecular basis for its high-affinity binding. Conversely, PltBd2 features a valine residue at the corresponding position (Figs 6B, triangle, 6E and S12), limiting its binding capacity for Neu5Gc-terminated glycans, akin to PltB in typhoid toxin. Unlike PltC and ArtB, which possess a basal, spoon-shaped glycan-binding site, neither PltBd1 nor PltBd2 exhibit this structural feature (Fig 6B, dashed frame and Fig 6F, 6G), making them more structurally similar to PltB. The locations of D-G are annotated on the overall structure in Fig 6C.

## Discussion

The presence of multiple distinct B subunits within AB_5_ toxins has been observed in various human pathogens. For example, typhoid toxin in *S.* Typhi incorporates two distinct homopentameric B subunits, while pertussis toxin in *Bordetella pertussis* employs four different B subunits in a single heteropentameric structure [9]. These examples illustrate the structural and functional diversity of B subunits, though their physiological roles remain incompletely understood. In this study, we identify the diarizonae toxin from *S. diarizonae* as a key virulence factor and demonstrate that its holotoxin comprises a trimeric PltBd1 and a dimeric PltBd2, forming a heteropentameric structure. Our data suggest that these two B subunits perform distinct yet complementary roles: PltBd1 facilitate toxin secretion from infected cells, whereas PltBd2 primarily mediate host cell targeting (Fig 6H).

Our study reveals distinct carbohydrate-binding properties of PltBd1 and PltBd2. ITC analyses demonstrated that both PltBd1 and PltBd2 pentamers recognize Neu5Ac-terminated glycans, with PltBd2 exhibiting stronger Neu5Ac affinity. Notably, PltBd1 displays unique binding capacity toward Neu5Gc-terminated glycans, a capability absents in PltBd2. These differences likely reflect adaptation to distinct glycan environments, as *Salmonella* remodels the SCV membrane composition to promote intracellular survival [28], potentially creating different glycan receptors between SCV and host cell surfaces. The functional roles of PltB subunits are closely linked to their glycan-binding properties, as demonstrated by extensive experimental evidence [15,27]. Typhoid toxin’s human specificity stems from PltB’s exclusive Neu5Ac recognition [15], while *S*. Typhimurium toxin’s broader host range results from its B subunit ArtB’s dual Neu5Ac/Neu5Gc binding capacity [27]. In our study, experiments using two distinct cell types suggested that PltBd1 and PltBd2 preferentially mediate toxin secretion and cellular uptake, respectively, suggesting their distinct glycan affinities may drive functional specialization. We propose that PltBd1’s dual specificity ensures efficient toxin secretion across varying SCV glycan environments, while PltBd2’s higher Neu5Ac affinity optimizes host cell targeting. However, further validation is needed through more definitive glycan array analysis based on the identified critical features (i.e., α2-3-linked sialic acid and Galβ1-4Glc) and direct characterization of glycan profiles on SCV and host cell surfaces.

Analysis of the interaction interfaces between PltBd1 and PltBd2 revealed conserved polar interactions that likely contribute to the stable assembly of the heteropentamer. Additionally, interactions between PltA and the PltB subunits suggest that PltBd1 plays a role in narrowing the pentamer pore via spatial hindrance, thereby stabilizing polar interactions with the C-terminal tail of PltA. In contrast, PltBd2 supports stable hydrophobic interactions with PltA. Although PltBd1 and PltBd2 can individually form homopentamers, the heteropentamer is preferentially selected by PltA, likely due to its enhanced structural compatibility and complementary interactions. This finding underscores the functional and structural advantages of the heteropentameric assembly.

Sequence analysis indicates that PltBd1 and PltBd2 share greater similarity with PltB than with ArtB, suggesting that these subunits may represent evolutionary intermediates in the transition from early ArtB to the more advanced PltB [49,50]. Furthermore, the unique genomic arrangement of *pltBd1* and *pltBd2* within a single genomic island was observed not only in *S. enterica* ssp. *diarizonae* but also in other *Salmonella enterica* subspecies—including ssp. *enterica*, ssp. *salamae*, and ssp. *arizonae* (S13–S15 Figs)—indicates that the heteropentameric assembly is a widespread phenomenon in *Salmonella*. These findings highlight the evolutionary significance of heteropentameric toxin assembly, emphasizing its role in diversifying A_2_B_5_ toxins and advancing our understanding of their evolutionary trajectory and functional diversity.

In conclusion, this study reveals the pivotal role of the diarizonae toxin in the pathogenicity of *S. diarizonae*, marking a significant step forward in understanding bacterial virulence mechanisms. The heteropentameric assembly of PltBd1 and PltBd2, with their distinct but complementary roles in toxin secretion from infected cells and toxin endocytosis into host cells, provides critical insights into the molecular mechanisms underlying A_2_B_5_ toxin assembly and functional specialization. These findings open avenues for further research into bacterial toxin biology and lay the groundwork for developing targeted therapeutic strategies to counteract bacterial infections.

## Materials and methods

### Bacterial strains and cell lines

The *S. diarizonae* strains utilized in this study were derived from the wild-type isolate GS270 and modified through standard recombinant DNA techniques combined with allelic exchange, employing *E. coli* β-2163 Δ*nic35* as the conjugative donor strain [51]. A detailed list of bacterial strains used is provided in S1 Table. For routine culture, strains were grown in LB broth at 37 °C. A typhoid toxin-inducing medium (TTIM) was employed to mimic the SCV environment, facilitating toxin secretion into the medium, containing 0.2% glucose as a carbon source, supplemented with the indicated concentrations of MgCl_2_ and adjusted to pH 7.2 [52]. Normal human colon mucosal epithelial (NCM460) cells were cultured in RPMI 1640 medium supplemented with 10% fetal bovine serum and 1% penicillin-streptomycin (complete medium) [31]. Cells were maintained at 37 °C in a 5% CO₂ incubator, with subculturing performed at a 1:3 ratio every three days. Cells were refreshed after reaching 20 passages, and mycoplasma contamination was routinely monitored using a mycoplasma detection kit.

### Plasmid construction

All plasmids used in this study are detailed in S2 Table. For recombinant protein expression in *E. coli*, *pltBd1*, *pltBd2*, *pltA*, and *cdtB* were cloned into the pET28b (Novagen) vector or cloned into the pACYC (Novagen) vector by inserting each of the subunits as a single operon in pET28b or pACYC [52,53]. For *in situ* genetic manipulation in *Salmonella*, ~ 1000 bp overlap upstream and downstream of the target region were cloned into pSB890 vector [54]. For *S. diarizonae* Δ*pltBd1d2* complementation studies, we used plasmid pBAD, which encodes an arabinose-inducible promoter. All plasmids were constructed using Gibson assembly [55] and verified by DNA sequencing.

### Protein expression and purification

For *in vitro* recombination protein expression, *E. coli* BL21 (DE3) carrying a plasmid encoding the Strep-DrICE-CdtB/Strep-DrICE-PltBd1/Strep-DrICE-PltBd2 construct was cultured in 2 L of LB medium to an optical density at 600 nm (OD_600_) of 0.8. Protein expression was induced by addition 0.3 mM isopropyl-β-d-thiogalactopyranoside (IPTG), followed by incubation for 12 h at 18 °C. For *in situ* protein expression, overnight cultures of the *S. diarizonae* strain expressing Strep-DrICE-CdtB were grown in 12 L of TTIM for 24 h.

Bacterial cells were harvested by centrifugation, and the pellets were resuspended in 15 mL lysis buffer (20 mM Tris-HCl, pH 8.0, 150 mM NaCl) per liter of culture. Bacterial cells were lysed using a high-pressure cell crusher (Union-Biotech). The supernatants were collected, run through Strep-Tactin Beads (Smart-Lifesciences). After washing with lysis buffer, the DrICE tag was removed with homemade DrICE protease at 25 °C for 3 h. The proteins were further purified by ion-exchange chromatography using a HiTrap Q column (GE Healthcare, Cat No. 17115401). The resulting fractions were analyzed by sodium dodecylsulfate (SDS)–polyacrylamide gel electrophoresis (PAGE) and further purified by Superdex 200 Increase (GE Healthcare, Cat No. 28990944).

### *Salmonella diarizonae* infections

To synchronize the cell cycle, cells were passaged once prior to the experiment. In the next day, cells were harvested and seeded in 12-well plates at a density of 3 × 10^^4^ cells per well. After 24 h, overnight-cultured *Salmonella* bacteria were diluted to an OD_600_ of 0.8 in fresh LB medium containing 0.3 M NaCl at a ratio of 1:300. Cells were infected with bacteria at a specified Multiplicity of Infection (MOI) for 1 h in Hank’s Balanced Salt Solution (HBSS). Following infection, cells were washed three times with PBS and then incubated for 1 h in the culture medium containing 100 μg/mL gentamicin to eliminate extracellular bacteria. After two additional PBS washes, cells were incubated in the culture medium containing 30 μg/mL gentamicin. Cell collection was performed 24/60 h post-infection.

### Culture cell intoxication assays

To evaluate the cytotoxicity of diarizonae toxin in cultured human cells, NCM460 cells were seeded in 12-well plates, cultured for 24 h, and then exposed to fresh medium containing the desired toxin concentration. Following medium replacement, cells were directly cultured, and cell collection occurred 60 h later. To calculate EC50 values, the percentage of cells in the G2/M phase (% G2/M) for each sample was determined and normalized by subtracting the % G2/M of untreated cells, then divided by the difference between the maximum % G2/M (set at 90% based on consistent observations at saturating toxin levels) and the % G2/M of untreated cells.

### Cell viability assay

To assess the toxicity of the bacteria/toxin in human cells, we quantified the number of cells arrested in the G2/M phase (indicative of CdtB-mediated DNA damage) using flow cytometry, as previously described [15]. Briefly, the cells were removed from the dishes using trypsin treatment, pelleted, re-suspended in 300 µL PBS, fixed by adding 700 µL prechilled ethanol and incubating overnight at -20 °C. The fixed cells were pelleted, washed with PBS, and then incubated with PBS containing 50 μg/mL propidium iodide, 0.1 μg/mL RNase A, and 0.05% Triton X-100 for 40 min at 37 °C. Cells were then pelleted, washed, resuspended in PBS, filtered, and analyzed by a flow cytometer (BD, Cat No. C6). Flow cytometry data were analyzed using FlowJo software.

### Quantitative reverse transcriptase real-time PCR

For RT-qPCR analyses, bacteria were harvested after cultivation in 6 mL of TTIM for approximately 7 h, reaching an OD at 600 nm of 0.9. The harvested cells were frozen in liquid nitrogen and stored at -80 °C until further analysis. Total RNA extraction was performed using the Genstone Biotech RNA extraction Kit (Cat No. TR214-D-50) following the manufacturer’s instructions. RNA quality and quantity were assessed using the ND-1000 NanoDrop spectrophotometer. Reverse transcription was carried out with the Takara cDNA Synthesis Kit (Cat No. RR047A).

Quantitative reverse transcriptase real-time PCR was conducted using the Takara TB Green Premix EX Taq Ⅱ (Cat No. RR820A). The amplification was performed in a total volume of 20 µL, including 10 µL of 2 × TB green Premix, 2 µL of cDNA (80 ng), and 1 µL of each specific primer. The sequences of primers were list in S3 Table. A master mix was prepared for three biological replicates and three technical replicates for each sample. The thermal cycling protocol involved initial denaturation at 95 °C for 10 min, followed by 40 cycles of denaturation at 95 °C for 15 s, primer annealing at 60 °C for 30 s, and elongation at 72 °C for 30 s. A negative control reaction was included for each primer set by omitting template cDNA. The comparative CT method (2^−∆∆CT^ method) was utilized to analyze the expression levels of the target genes [56].

### Western blotting analysis

*S. diarizonae* and its mutant bacteria were grown overnight in LB, washed twice using TTIM, diluted 1/100 into 5 mL of fresh TTIM, and grown overnight. The strain pellets were analyzed by SDS-PAGE and transferred to polyvinylidene difluoride membranes (Millipore). Membranes were blocked with 5% skimmed milk solution and treated with the primary antibodies (Rabbit anti-DnaK, Cusabio, Cat No. CSBPA633459HA01EGW, 1:2,500; Mouse anti-FLAG, MBL, Cat No. M180-3, 1:10,000; Mouse anti-Strep, MBL, Cat No. M211-3, 1:5,000) and horseradish peroxidase (HRP)-conjugated secondary antibody (Goat anti-rabbit, MBL, Cat No. 458, 1:5,000; Goat anti-mouse, MBL, Cat No. 330, 1:5,000). Blots were developed with M5 HiPer ECL Western HRP Substrate (Mei5bio, Cat No. MF074).

### Co-immunoprecipitation analysis

To identify interaction partners for various diarizonae toxin subunits, *S. diarizonae* and its mutant bacterial cell lysates were immunoprecipitated using C-terminal Strep/HA epitope-tagged PltBd1/CdtB or His epitope-tagged PltBd2 (tags incorporated at the native genomic locus), as indicated, and the eluates were analyzed by western blot as is described above. For *in vitro* grown samples, the indicated strains were grown overnight in LB, washed twice using TTIM, diluted 1/100 into 200 mL of fresh TTIM, and grown overnight. For *in vivo* grown samples, six 15 cm dishes containing a total of 9 × 10^^7^ NCM460 cells were infected at an MOI of 50 as described above using each of the indicated *S. diarizonae* strains and cultured for 24 h. For each sample, cells were pelleted, resuspended in lysis buffer (50 mM Tris pH 7.5, 150 mM NaCl, protease inhibitors mixture [Solarbio, Cat No. P6730], 40 µg/mL DNase I), and crushed by a high-pressure cell crusher (Union-Biotech) at 4 °C. Cell lysates were prepared by high-speed centrifugation (17,000 × *g*, 50 min, 4 °C), and clarified lysates were immunoprecipitated for 2 h at 4 °C using different resin. Immunoprecipitated samples were thoroughly washed using lysis buffer and eluted using 10 mM desthiobiotin for Strep resin, 0.2 M glycine for HA resin, or lysis buffer containing 300 mM imidazole for Ni resin.

### Expression and purification of toxin proteins

The genes encoding PltBd1 or PltBd2 were individually cloned into the pET28b plasmid, which also harbored the *pltA* gene and the *cdtB* gene with a C-terminal DrICE-Strep tag. The plasmids were transformed into *E. coli* BL21 (DE3) strain. For the heteropentameric toxin, the pACYC plasmid carrying the *pltBd2* gene and the pET28b plasmid carrying the *pltBd1, pltA, and cdtB* genes with a C-terminal DrICE-6*His tag on PltBd2 and a C-terminal DrICE-Strep tag on CdtB, respectively, were co-transformed into *E. coli* BL21 strain. Cultures were grown in LB medium at 37 °C with shaking at 220 rpm until reaching an OD_600_ of approximately 0.8. Induction was initiated by adding 0.4 mM IPTG, followed by incubation at 25 °C for 18 h. Subsequently, cells were centrifuged to collect the bacterial pellet, which was then lysed in a buffer containing 20 mM Tris and 15 mM NaCl. The lysate was centrifuged at 4 °C for 50 minutes at 20,000 × *g*. After collecting the supernatant, Strep tag and His tag were employed for affinity chromatography. The released proteins were further purified by HiTrap Q HP ion-exchange chromatography and Superdex 200 Increase 10/300 GL gel filtration chromatography in an AKTApure FPLC chromatography system.

Heteropentameric toxins extracted *in situ* from the genome utilized TTIM, employing a chemically defined growth medium based on N minimal medium, as previously described. The subsequent steps for protein purification mirrored those described for the *E. coli*-derived toxin.

### Cryo-EM grid preparation and data collection

The cryo-electron microscopy grids for all protein samples were meticulously prepared using a Vitrobot Mark IV (FEI) at 8 °C under 100% humidity conditions. A 4 μL aliquot of the sample, with a concentration ranging from 6 to 8 mg/mL, was delicately applied to glow-discharged holey carbon grids (Au200 mesh, Quantifoil R 2/1). The grid preparation involved a 10-second wait time, a 4-second blot time, and a blot force of 1, followed by rapid freezing in liquid ethane.

Subsequently, the qualified grids were meticulously transferred to a Titan Krios microscope (FEI) operating at 300 kV and equipped with an energy filter (slit width 20 eV; GIF Quantum LS, Gatan) for data acquisition. Images were precisely recorded using a K3 Summit direct electron detector (Gatan) in super-resolution mode at a nominal magnification of ×81,000, corresponding to a calibrated pixel size of 0.52 Å. The data acquisition process was automated using EPU software, maintaining an electron dose of 60e/Å2.

### Cryo-electron microscopy image processing

For the genomic *in situ*-extracted PltBd1/PltBd2 heteropentameric holotoxin, 5,376 movies were processed starting with motion correction (MotionCorr2) and CTF correction (CTFFIND4). After the removal of poor-quality micrographs, 4,954 micrographs were retained for further processing. Automatic particle picking was performed using Blob picker on 50 micrographs, followed by 2D classification. Representative averages of protein projections in different orientations were selected as templates, and a total of 5,043,698 particles were picked based on the templates. These extracted particles underwent two rounds of re-extraction and bin2 processing. After 2D classification, 3,453,359 particles with superior 2D averages were selected. Six rounds of ab initio reconstruction and heterogeneous refinement were performed to enhance data quality. Finally, 1,341,334 particles from the best class were used for 3D reconstruction by non-uniform (NU) refinement, resulting in a map with a resolution of 2.32 Å, with no symmetry imposed.

For the PltBd1 homopentameric holotoxin from *E. coli*, 5,091 movies were processed similarly to the genomic *in situ*-extracted heteropentameric toxin, with motion correction and CTF correction. After automatic particle picking, two rounds of 2D classification were performed, followed by six rounds of ab initio reconstruction and heterogeneous refinement, resulting in 3,787,818 particles. The final non-uniform (NU) refinement resulted in a map at 3.05 Å resolution.

For the PltBd2 homopentameric holotoxin from *E. coli*, 1,670 movies were processed similarly, starting with motion correction and CTF estimation. After automatic particle picking and two rounds of 2D classification, four rounds of ab initio reconstruction and heterogeneous refinement were applied, resulting in 2,868,630 particles. The final non-uniform (NU) refinement produced a map at 2.61 Å resolution.

For the PltBd1/PltBd2 heteropentameric holotoxin from *E. coli*, 5,763 movies were processed in the same manner as the PltBd1 homopentameric toxin. The final non-uniform (NU) refinement resulted in a map with a resolution of 2.94 Å.

Detailed data collection parameters and processing workflows are provided in the S2–S5 Figs. and S4 Table.

### Model building and structural analysis

The CryoNet online model-building software was utilized for constructing all protein density maps. In cases where the software did not complete the density maps, meticulous manual adjustments were carried out in COOT to derive the final atomic models of the proteins. Model refinement was conducted using the real_space_refine module of PHENIX [57], incorporating secondary structure and geometric constraints to prevent overfitting. Following manual adjustments in COOT [58], the model underwent additional real-space refinement in PHENIX. Detailed statistics on cryo-EM data collection and refinement can be found in the supplementary materials, S4 Table. For structural analysis and visualization, all models were examined using UCSF Chimera and PyMOL. In particular, structural alignments were performed using the align command in PyMOL to compare homologous models and assess conformational differences. Interatomic distances between key residues or domains were also measured in PyMOL to aid in functional interpretation.

### Visualization of toxin export carrier intermediates

The visualization of diarizonae toxin vesicle carrier intermediates was performed as previously described [23]. Briefly, 24 h post-infection with the indicated *S. diarizonae* strains expressing FLAG epitope-tagged CdtB, cells were fixed in 4% paraformaldehyde, treated with 50 mM NH_4_Cl for 10 min, and then blocked with PBS containing 3% BSA and 0.3% Triton X-100. For immunostaining, fixed cells were incubated with primary mouse monoclonal anti-FLAG (MBL) and rabbit polyclonal anti-*Salmonella* antibodies (Abcam), followed by Alexa 488-conjugated anti-mouse and Alexa 647-conjugated anti-rabbit antibodies (Cell signaling technology). Samples were visualized under a Zeiss LSM900 laser scanning confocal microscope.

### Isothermal titration calorimetry

To evaluate the binding affinity between glycans and the B subunits, isothermal titration calorimetry (ITC) was performed with a Malvern ITC device. Stock solutions of Neu5Ac*α*2-3G*a*l*β*1-4Glc (3Ac) or Neu5Gc*α*2-3Gal*β*1-4Glc (3Gc) at 100 mM were diluted into 15 mM using ITC buffer (20 mM HEPES, pH 7.5, and 150 mM NaCl). Purified PltBd1 and PltBd2 pentamers were exchanged into ITC buffer with a Superdex 200 Increase column. For each titration, 300 μL of B subunit (70 μM) was titrated with 70 μL of glycan solutions in 19 steps at 25 °C. The resulting data were analyzed using the MicroCal evaluation software (Malvern, Worcestershire, UK).

### Statistical analysis

At least three independent biological replicates were performed for each experiment, yielding consistent results. Data are presented as the arithmetic mean ± standard deviation (s.d.). Statistical analyses were performed using GraphPad Prism v.9.3.0 (GraphPad). For comparisons of gene transcription levels of the same bacterial strain under different culture conditions (e.g., LB medium vs. TTIM medium), a paired two-tailed Student’s t-test was applied. Normality was confirmed using the Shapiro-Wilk test (*p* > 0.05). For other comparisons, unpaired two-tailed Student’s *t*-*t*ests were employed to analyze the statistical significance between two groups. Normality was verified with the Kolmogorov-Smirnov test (*p* > 0.05). The significance of mean comparisons was annotated as follows: *p < 0.05; **p < 0.01; ***p < 0.001; ****p < 0.0001; ns indicates no significant difference. Statistical significance was defined as *p* < 0.05. No statistical method was used to predetermine sample size. No data were excluded from the analyses. The experiments were not randomized. The Investigators were not blinded to allocation during experiments and outcome assessment.

## Supporting information

S1 Fig Different tests for verifying the indispensable roles of PltBd1 and PltBd2. **(A)** Amino acid sequence alignment of CdtB/PltA and its homologous subunit. Conserved residues are highlighted in red. The pentacle marks the conserved active catalytic site (His160) in CdtB. The triangle indicates the conserved disulfide bond connecting CdtB to PltA. **(B-C)** The histograms show the normalized mean intensity (with standard deviations [s.d.]) of the CdtB, PltBd1 and PltBd2 bands from the samples (Fig 1D and 2A) in three independent experiments. *p < 0.05, **p < 0.01, ***p < 0.001. **(D)** Cytotoxicity assays showing the loss of cytotoxicity in Δ*pltBd1*, Δ*pltBd2,* and Δ*pltBd1d2* mutant strains towards NCM460 cells (MOI = 10). The proportion of cells in G2/M, reflecting the toxic activity of CdtB, following infection with wild-type *S. diarizonae* and its mutant strains, was determined by flow cytometry. An unpaired two-tailed *t*-test was used *t*o analyze the statistical significance; ns (not significant), ****p < 0.0001. Data in experiment **D** represent the mean ± s.d. of at least three independent evaluations with triplicate measurements per replicate. **(E-F)** Western blot and RT-qPCR analyses showing the deletion of *pltBd1* influenced the transcription and expression levels of PltBd2 subunit. Experiments were conducted at least three times with consistent results. Statistical analysis was performed using paired and unpaired two-tailed *t*-tests; *p < 0.05. **(G)** Workflow for purification of *t*he PltBd1/PltBd2 heteropentameric holotoxin recombinantly expressed in *E. coli*, employing Strep-Tactin and Ni-NTA affinity chromatography and gel filtration. Created in BioRender. Gao, X. (2025) https://BioRender.com/o05m2ra **(H)** Purification and verification of the PltBd1/PltBd2 heteropentameric holotoxin recombinantly expressed in *E. coli*.
(TIF)

S2 Fig Cryo-EM structural determination of the *in situ*-purified PltBd1/PltBd2 heteropentameric holotoxin. **(A-C)** Purification and structural analysis process of the PltBd1/PltBd2 heteropentameric holotoxin expressed in *S. diarizonae in situ*. **(D)** The local resolution distribution of the PltBd1/PltBd2 heteropentameric holotoxin was calculated with cryoSPARC and presented in Chimera. **(E)** Angular distribution of the particles used for reconstruction of the holotoxin. **(F)** Gold-standard Fourier Shell Correlation (FSC) curves of the holotoxin. **(G)** Representative Cryo-EM densities in the PltBd1/PltBd2 heteropentameric holotoxin. The densities, contoured at 5–8 σ, were prepared in PyMOL.
(TIF)

S3 Fig Cryo-EM structural determination of the PltBd1 homopentameric holotoxin recombinantly expressed in *E. coli.* **(A-B)** Structural analysis process of the PltBd1 homopentameric holotoxin recombinantly expressed in *E. coli*. **(C)** Angular distribution of the particles used for reconstruction of the holotoxin. **(D)** Gold-standard FSC curves of the holotoxin.
(TIF)

S4 Fig Cryo-EM structural determination of the PltBd2 homopentameric holotoxin recombinantly expressed in *E. coli.* **(A-B)** Structural analysis process of the PltBd2 homopentameric holotoxin recombinantly expressed in *E. coli*. **(C)** Angular distribution of the particles used for reconstruction of the holotoxin. **(D)** Gold-standard FSC curves of the holotoxin.
(TIF)

S5 Fig Cryo-EM structural determination of the PltBd1/PltBd2 heteropentameric holotoxin recombinantly expressed in *E. coli.* **(A-B)** Structural analysis process of the PltBd1/PltBd2 heteropentameric holotoxin recombinantly expressed in *E. coli*. **(C)** High-resolution Cryo-EM reconstruction of the PltBd1/PltBd2 heteropentameric holotoxin, revealing the pseudo-symmetric arrangement of PltBd1 and PltBd2 subunits in a 3:2 ratio. **(D)** The local resolution distribution of the PltBd1/PltBd2 heteropentameric holotoxin was calculated with cryoSPARC and presented in Chimera.
(TIF)

S6 Fig Close-up views of Cryo-EM electron density maps highlighting the amino acid markers used to distinguish PltBd1 and PltBd2. **(A)** Close-up views of Cryo-EM electron density maps highlighting the amino acid markers used to distinguish PltBd1 and PltBd2. **(B)** Structural alignment of holotoxin from *S. diarizonae* (cyan) and *E. coli* (yellow) obtained by single-particle Cryo-EM.
(TIF)

S7 Fig Structural alignment of PltB^*S.Ty*^, PltBd1, and PltBd2. The overall structures of PltB^*S.Ty*^, PltBd1, and PltBd2 are highly similar. Close-up views show the structurally conserved locations of three key regions: The disulfide bond-forming cysteines (PltB^*S.Ty*^: C133/C128; PltBd1: C113/C107; PltBd2: C110/C105); The second disulfide bond-forming cysteines (PltB^*S.Ty*^: C70/C54; PltBd1: C49/C32; PltBd2: C47/C31); The serine residue (PltB^*S.Ty*^: S35; PltBd1: S13; PltBd2: S12). Despite differences in amino acid identity, the protein backbone in these regions is nearly identical, supporting a conserved structural framework.
(TIF)

S8 Fig Co-immunoprecipitation (Co-IP) analyses. **(A)** Functional validation of interfacial residues in pentamer assembly. Site-directed mutagenesis was performed on nine conserved residues located at the PltBd1/PltBd2 interface (mutant set M9: PltBd1-E1A, W2A, Q24A, Y30H, S60A, N67A, R69A, Y72H, N76A; PltBd2-E1A, W2A, Q23A, Y29H, S58A, N65A, R67A, Y70H, H74A). Co-immunoprecipitation assays revealed that site-directed mutagenesis of interfacial residues disrupts PltB heteropentamer assembly. **(B)** Co-IP analysis of the homopentameric or heteropentameric assembly purified from wild-type and *in situ* knockout strains. PltB forms stable pentamers independently of PltA and CdtB. Experiments **A-B** were conducted at least three times with consistent results.
(TIF)

S9 Fig The interaction between the C-terminal tail of PltA and B subunits in homo- and hetero-pentameric holotoxins. (TIF)

S10 Fig (A) Relative toxin puncta-associated fluorescence intensity. Data from confocal images in Fig 5E. **p < 0.01. **(B)** Co-IP analysis of holotoxins purified from wild-type and complemented strains. Complementation with both *pltBd1*/*pltBd1*^*S35A*^ and *pltBd2/pltBd2*^*S35A*^ resulted in the assembly of PltBd1/PltBd2 heteropentameric holotoxin. Experiment **B** was conducted at least three times with consistent results.
(TIF)

S11 Fig Distinct and synergistic functions of PltBd1 and PltBd2 in toxin secretion and toxin endocytosis, performed with human colon adenocarcinoma cell line HT-29. (A) Cytotoxicity of wild-type and S35A-mutated PltBd1/PltBd2 homopentameric holotoxins was measured in HT-29 cells. (B-C) Cytotoxicity of wild-type and S35A-mutated PltBd1/PltBd2 heteropentameric holotoxins was measured in HT-29 cells. Data in histogram represents the mean ± s.d. of three biological replicates (n = 3), each performed in triplicate. (D) Immunofluorescence imaging of toxin transport carriers in HT-29 cells infected with various *S. diarizonae* strains expressing FLAG-tagged CdtB. Discrete CdtB puncta, representing toxin release from SCVs, were detected using a mouse anti-FLAG monoclonal antibody (green). *Salmonella* and HT-29 were visualized using a rabbit anti-*Salmonella* antibody (red), and DAPI nuclear staining (blue), respectively. Confocal laser scanning microscopy revealed toxin localization within the host cells. Scale bar: 10 µm. (E-F) Cytotoxicity assays performed on HT-29 cells infected with *S. diarizonae* strains complemented with heteropentameric holotoxins (MOI = 10). Data in histogram represents the mean ± s.d. of three biological replicates (n = 3), each performed in triplicate. Statistical significance was analyzed using unpaired two-tailed *t*-tests; ns, not significant, ****p < 0.0001.
(TIF)

S12 Fig Structural basis for differential Neu5Gc recognition by PltBd1 and PltBd2. The superposition of PltBd1 (green) and PltC (blue, PDB:7EE5) reveals that the phenolic hydroxyl group of Tyr103 forms a 2.9 Å hydrogen bond with the hydroxyl group of Neu5Gc (black dashed line). In contrast, the Val of PltBd2 (purple) exhibits a significant gap with Neu5Gc, thereby hindering effective binding.
(TIF)

S13 Fig Phylogenetic tree of *Salmonella* strains harboring *pltBd1* and *pltBd2* within the same diarizonae toxin gene cluster. (TIF)

S14 Fig Amino acid sequence alignment of PltBd1 from strains listed in S13 Fig with its homologous proteins PltB, ArtB, and PltC. (TIF)

S15 Fig Amino acid sequence alignment of PltBd2 from strains listed in S13 Fig with its homologous proteins PltB, ArtB, and PltC. (TIF)

S1 Table List of bacterial strains used in this study. (XLSX)

S2 Table List of plasmids used in this study. (XLSX)

S3 Table Primer sequences used in this study for RT-qPCR. (XLSX)

S4 Table Cryo-EM data collection and refinement statistics. (XLSX)
